# The optical coherence tomography and microperimetry biomarker evaluation in patients with geographic atrophy (OMEGA) study: Geographic atrophy progression in fundus autofluorescence ‐ OMEGA report 3

**DOI:** 10.1111/aos.70119

**Published:** 2026-03-08

**Authors:** Philippe Valmaggia, Georg Ansari, Nadja Inglin, Nils Schärer, Hanna Zuche, Chrysoula Gabrani, Faady Yahya, Marco Cattaneo, Kristina Pfau, Andrea Giani, Marieh Esmaeelpour, Taffeta Chingning Yamaguchi, Christian Prünte, Hendrik P. N. Scholl, Nicolas Feltgen, Leopold Schmetterer, Maximilian Pfau, Peter M. Maloca

**Affiliations:** ^1^ Department of Ophthalmology University Hospital Basel Basel Switzerland; ^2^ Department of Biomedical Engineering University of Basel Allschwil Switzerland; ^3^ Institute of Molecular and Clinical Ophthalmology Basel (IOB) Basel Switzerland; ^4^ Department of Clinical Research University of Basel Basel Switzerland; ^5^ Boehringer Ingelheim International GmbH Ingelheim am Rhein Germany; ^6^ Vue Eye Center Biel/Bienne Switzerland; ^7^ Pallas Kliniken AG, Pallas Klinik Zürich Zürich Switzerland; ^8^ European Vision Institute Basel Switzerland; ^9^ Department of Clinical Pharmacology Medical University of Vienna Vienna Austria; ^10^ Singapore Eye Research Institute, Singapore National Eye Centre Singapore Singapore; ^11^ Academic Clinical Program Duke‐NUS Medical School Singapore Singapore; ^12^ SERI‐NTU Advanced Ocular Engineering (STANCE) Programme Singapore Singapore; ^13^ Center for Medical Physics and Biomedical Engineering Medical University of Vienna Vienna Austria; ^14^ School of Chemistry, Chemical Engineering and Biotechnology Nanyang Technological University Singapore Singapore; ^15^ Lee Kong Chian School of Medicine Nanyang Technological University Singapore Singapore; ^16^ Moorfields Eye Hospital NHS Foundation Trust London UK

**Keywords:** age‐related macular degeneration, disease progression, fundus autofluorescence, geographic atrophy, natural history

## Abstract

**Purpose:**

To measure the progression of geographic atrophy (GA) in subjects with age‐related macular degeneration (AMD) in a natural history study.

**Methods:**

We analysed fundus autofluorescence (FAF) images using a semiautomatic method (RegionFinder) to quantify the GA area in a monocentric study. Three independent graders analysed each eye. Patient visits were scheduled at baseline, 12, 24 and 48 weeks. The primary endpoint was the progression of GA area per eye. Potential correlations with clinical and imaging‐based parameters were analysed.

**Results:**

The annual GA progression rate was 1.16 mm^2^/year (95% CI: 0.76–1.56; *p* < 0.001), with faster growth in eyes with larger baseline lesions (*R* ^2^ = 0.74). After square root transformation, the progression rate was 0.34 mm/year (95% CI: 0.26–0.43; *p* < 0.001), and the size dependency was reduced (adjusted *R* ^2^ = 0.16). Multivariable mixed‐effects models identified a significant association between larger GA lesion sizes per eye and the number of smoking pack‐years (*p* = 0.017), the presence of perilesional hyperautofluorescence (*p* = 0.044) and a multifocal configuration of the GA lesions (*p* = 0.038). Intermediate AMD in the fellow eye was significantly associated with faster GA progression (*p* = 0.041).

**Conclusion:**

GA progression in AMD is significantly influenced by baseline lesion size and a larger number of smoking pack‐years is associated with larger GA lesions. The marked variability between individuals underscores the multifactorial nature of GA dynamics. Quantitative FAF analysis is a reliable method for assessing lesion progression and may inform risk‐based monitoring strategies.

## 1 INTRODUCTION

Geographic atrophy (GA), the advanced stage of age‐related macular degeneration (AMD), represents a leading cause of severe vision loss in older individuals (Lim et al., 2012; Wu et al., 2022). This condition is characterised by progressive degeneration of the outer retina and choriocapillaris over time, with foveal involvement serving as a critical determinant for the deterioration of visual function (Fleckenstein et al., 2024). Traditionally, visual function is assessed using best‐corrected visual acuity (BCVA); however, this metric does not capture other parameters that may significantly impact vision, especially in GA (Chan et al., 2024; Schmetterer et al., 2023). The ‘Optical Coherence Tomography and Microperimetry Biomarker Evaluation in Patients with Geographic Atrophy Study (OMEGA)’ is a study that aimed to systematically compare a panel of visual function and structural outcome measures for monitoring the progression of GA (ClinicalTrials: NCT05963646) (Ansari et al., 2023).

Fundus Autofluorescence (FAF) has emerged as the main imaging modality for measuring the progression of GA, yet particularly in cases of foveal sparing, optical coherence tomography (OCT) and scanning laser ophthalmoscopy (SLO) can provide valuable complementary information (Lindner et al., 2015; Pfau et al., 2023). FAF utilises the natural fluorescence of lipofuscin, a byproduct of photoreceptor metabolism that accumulates in the retinal pigment epithelium (RPE) cells (Schmitz‐Valckenberg et al., 2008). In GA, the loss of RPE cells leads to reduced autofluorescence, enabling the delineation of atrophic areas, making the technology particularly effective in detecting and quantifying the size of GA lesions. FAF is non‐invasive and does not require the injection of contrast agents, making it safer and more comfortable for patients during repeated examinations. Making measurements over time allows quantification of the progression in this disease, and characteristic FAF patterns have been shown to be associated with varying progression rates (Egger et al., 2025).

Alleviating the burden of AMD would benefit a large number of patients, particularly as its global prevalence continues to rise, especially in countries with ageing populations (Rein et al., 2009). A thorough understanding of the disease's natural progression is essential for assessing its trajectory and evaluating the effectiveness of potential treatments. Distinguishing between fast and slow progressors has been a focal point of research, with particular attention to cases involving foveal sparing—a condition where central vision remains preserved despite surrounding atrophy. Studies like the OMEGA study play a crucial role in advancing our understanding of lesion progression and the broader implications of novel treatments aimed at slowing disease progression. However, the mechanisms underlying lesion development remain incompletely understood, underscoring the need for further research, especially to evaluate the impact of emerging therapeutic strategies.

In this report, we investigated GA progression with FAF. Specifically, we analysed the progression of the total GA area per eye, examined changes in the number of individual lesions and assessed lesion confluence over time. Finally, the effect of clinical and FAF imaging‐based parameters on GA progression was analysed with multivariable regression models.

## 2 MATERIALS AND METHODS

### 2.1 Study design

The OMEGA study is an observational, prospective, natural‐history study. Ethical approval was obtained from the Ethics Committee of Northwestern and Central Switzerland (BASEC ID: 2019‐02003) and the study was performed according to the Declaration of Helsinki. Study enrolment took place at the Eye Clinic of the University Hospital Basel between March 2021 and July 2022, and written informed consent was obtained from all participants. The study included a baseline visit (V1) and follow‐ups at 12 (V2), 24 (V3) and 48 weeks (V4). Study visits took place between March 2021 and June 2023. Further details about the study protocol and the characteristics of the cohort, can be found in previous study reports (Ansari et al., 2023, 2025).

The study's primary aim is to systematically compare a panel of established and novel visual function and structural outcome measures for monitoring the progression of GA. These tests include best‐corrected visual acuity (BCVA), low‐luminance visual acuity (LLVA) and contrast sensitivity function evaluation. Imaging assessments include fundus photography, FAF, OCT and OCT angiography (OCTA). The main outcome variables for this analysis were the description of atrophy sizes as measured with FAF, the time‐resolved analysis of atrophy progression and the correlation of progression rates with clinical parameters.

### 2.2 Data sources/measurements

Short‐wavelength (blue) FAF images were captured using the Heidelberg Spectralis device (Heidelberg Engineering, Heidelberg, Germany) with a 30° × 30° field of view in HS mode, with an Automatic Real Time (ART) averaging of 20, and ART normalisation was enabled to ensure consistent intensity across the image. The image resolution was 768 × 768 pixels, corresponding to an en‐face field of approximately 9 mm × 9 mm. Imaging was performed in Automatic Real Time (ART) mode, with 20 images averaged to enhance quality. Macular imaging with spectral‐domain OCT (SD‐OCT) was performed using the same device, covering a 20° × 20° area (193 B‐scans) in HS mode with ART 16, approximately corresponding to a 6 mm × 6 mm region.

We used the RegionFinder software (Version 2.6.5.0, Heidelberg Engineering) to quantify lesion sizes in the FAF images. The software allows for individualised lesion grading by setting a seed point and adjusting an intensity‐based spreading for precise delineation of affected areas. Physiologically hypoautofluorescent areas such as the fovea and vessels, were blocked from spreading. In cases of uncertain foveal involvement, SD‐OCT and SLO images were consulted for confirmation. All images were graded independently by three graders. An example grading can be found in Figure 1.

**FIGURE 1:**
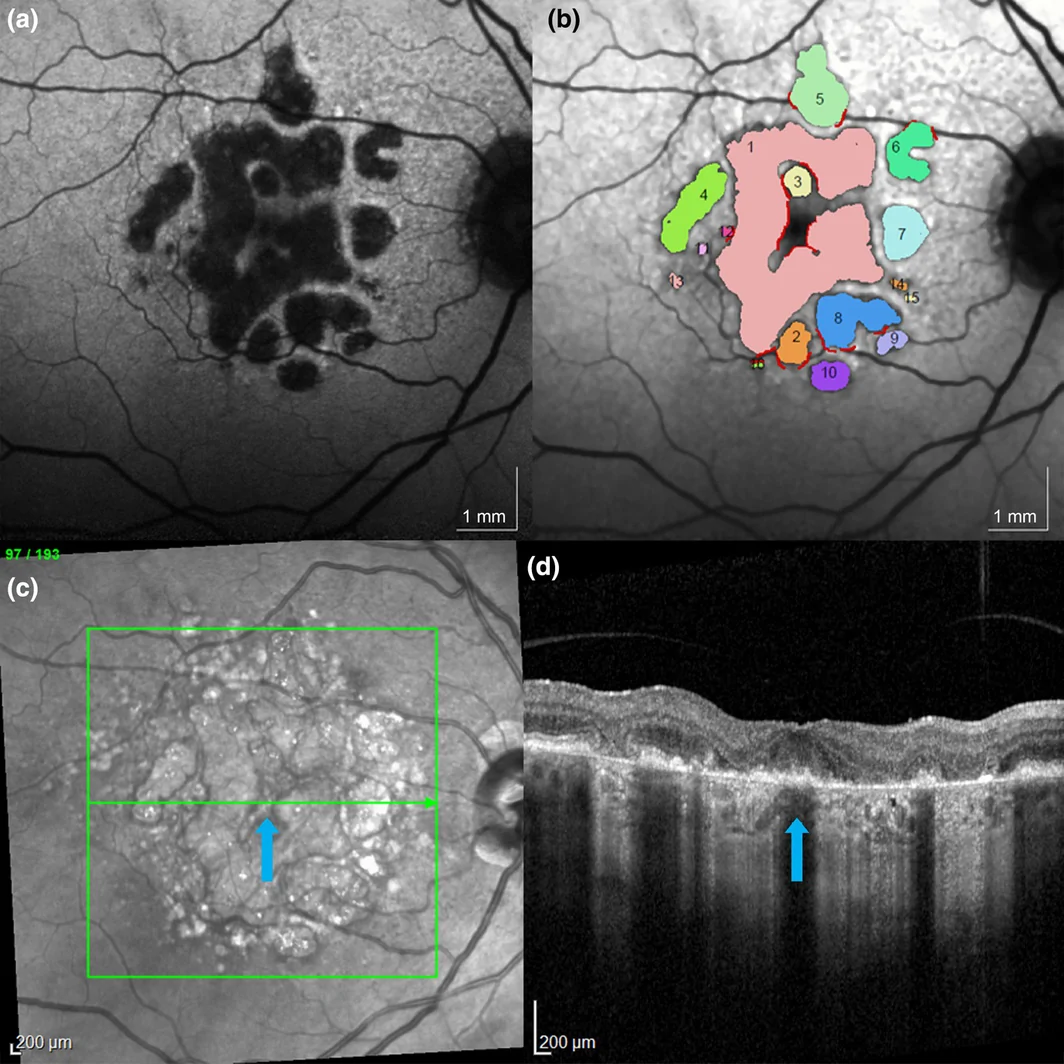
Geographic atrophy lesion annotation. (a) The original fundus autofluorescence (FAF) image. (b) The individual lesions annotated with RegionFinder, a semiautomatic annotation tool allowing for quantification of individual lesion sizes. (c) The corresponding scanning laser ophthalmoloscopy (SLO) and (d) optical coherence tomography (OCT) images were used to identify atrophy in case of uncertainties on the FAF image, such as foveal sparing.

### 2.3 Statistical analysis

The statistical analysis in this study involved both univariable and multivariable mixed‐effects models to evaluate the associations of various clinical and demographic variables with the outcomes of interest. The variables analysed included age at consent, sex, body‐mass‐index (BMI), smoking history (never, ex‐smoker, current smoker), smoking pack‐years (PY), lens status (pseudophakic, immature cataract (without surgery indication), mild cataract), best‐corrected visual acuity (BCVA), axial length and systolic blood pressure.

The study size was determined by the number of eligible individuals available during the recruitment period. No formal power calculation was conducted, as the study's exploratory nature focused on generating insights for future investigations. The inter‐grader agreement for lesion annotations was assessed using the intraclass correlation coefficient (ICC(2,1); two‐way random‐effects model with absolute agreement). Each eye‐visit combination was considered a measurement unit for ICC calculations. The progression rates were analysed for absolute values in mm^2^ as well as for the square‐root transformed values to reduce the dependence on the baseline area (Feuer et al., 2013). The progression rates were analysed between all visits and values from all graders were integrated into the mixed‐effects models, evaluating both clinical parameters and FAF imaging‐based variables. The final mixed‐effects model included a random slope and intercept at the patient level, and a random intercept for eye and grader, thereby accounting for the inclusion of both eyes from a subset of the study participants. To analyse the progression of GA, we performed regression analyses of the slope of progression against baseline values, complemented by multivariable regression models to account for both continuous and categorical covariates.

Univariable and multivariable regression tables were included to evaluate variables influencing GA progression. Statistical significance was set at a two‐sided p‐value <0.05. Continuous covariates were centred at 0, and the most common category was chosen as the reference for categorical covariates. This approach facilitates the interpretation of results: the global intercept and the ‘years’ slope correspond to the baseline value and annual progression when continuous covariates are at their mean and categorical covariates are at their reference category. For instance, the coefficients for ex‐smokers and smokers versus non‐smokers reflect the effects for individuals with the average number of pack‐years. For smokers without pack‐year information, the average number of pack‐years was taken. Also for non‐smokers, the average amount of pack‐years was chosen based on previous smoking models (Leffondré et al., 2002).

## 3 RESULTS

In the OMEGA study, 37 eyes of 30 patients were analysed. Thirty‐one eyes of 25 patients completed all four visits. Table 1 shows the study cohort characteristics. At the baseline visit, 112 lesions were identified in the 37 eyes; at visit 2, the same 37 eyes showed 114 lesions. By Visit 3, 34 eyes showed 114 lesions, indicating the appearance of new lesions in some eyes. At Visit 4, 31 eyes exhibited 106 lesions. Over the course of the study, five eyes developed new GA lesions, but no confluence of lesions was observed. The average number of lesions per eye increased slightly over time, from 3.03 ± 2.75 (mean ± standard deviation) at Visit 1 to 3.08 ± 2.73 at Visit 2, 3.32 ± 2.77 at Visit 3 and 3.39 ± 2.98 at Visit 4, reflecting the subtle dynamics of lesion emergence without evidence of merging. Overall inter‐grader agreement was very high, with an ICC(2,1) of 0.987 (*p* < 0.001, 95% CI 0.980–0.991). Two example trajectories from baseline to week 48 can be found in Figure 2.

**FIGURE 2:**
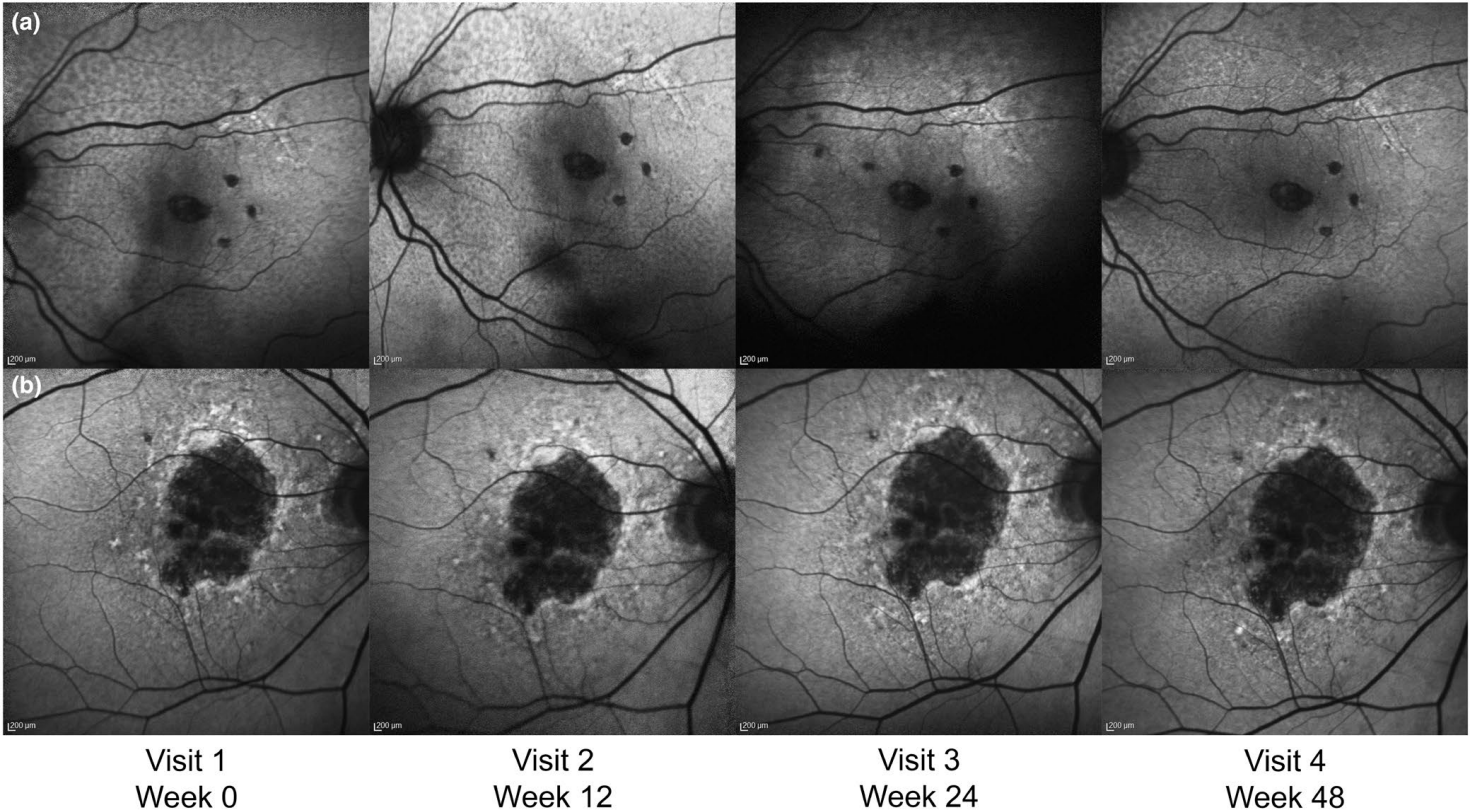
Progression of geographic atrophy (GA) areas. (a) Depiction of multiple small GA areas that showed minor progression. (b) Depiction of one large GA area that showed lateral progression and hyperautofluorescent signs of activity at the lesion border.

**TABLE 1:** Baseline demographic and clinical characteristics of the study cohort.

Variable	*n* = 30^a^
Age at consent	79 (66–90)
Gender	
Female	14 (47%)
Male	16 (53%)
Eye (*n* = 37)	
OD	19 (51%)
OS	18 (49%)
Ethnicity	
Caucasian	30 (100%)
Lens Status (*n* = 37)	
Immature cataract	4 (11%)
Mild cataract	6 (16%)
Pseudophakic	27 (73%)
Smoking status	
Ex‐smoker	13 (43%)
Never	12 (40%)
Smoker	5 (17%)
Height [cm]	167 (145–187)
Weight [kg]	72 (44–108)
BMI [kg/m^2^]	24.95 (17.81–34.47)
SBP [mmHg]	133 (96–183)
DBP [mmHg]	83 (57–111)

Abbreviations: BMI, body mass index; DBP, diastolic blood pressure; SBP, systolic blood pressure.

^a^ *n* = number of participants except for eye‐related variables, Median (min–max) for numerical data, *n* (% of total) for categorical data.

The analysis of GA area progression per eye in the OMEGA study with a linear mixed‐effects model showed an annual progression rate of GA area of 1.16 mm^2^/year (*p* < 0.001, 95% CI 0.76–1.56 mm^2^/year). The model accounted for random effects related to the subject (intercept and slope), eye (intercept) and grader (intercept). Further, the relationship between the slope of GA progression and baseline lesion size was investigated using linear regression. The analysis revealed a strong positive correlation, with a regression coefficient of 0.28 (*p* < 0.001), indicating that larger baseline lesions are associated with faster progression rates. The model had an *R* ^2^ value of 0.74, suggesting that 74% of the variability in lesion progression could be explained by baseline GA size alone. This finding underscores the prognostic significance of baseline lesion area in forecasting the future trajectory of GA growth and may have implications for stratifying patients in clinical trials or personalised monitoring strategies.

When applying a square root transformation to the GA area, the mixed model shows an annual progression rate of 0.34 mm/year (*p* < 0.001, 95% CI 0.26–0.43 mm/year). The relationship between baseline lesion size and progression rate was substantially reduced compared to the non‐transformed model (regression coefficient 0.09, adjusted *R* ^2^ = 0.16). The attenuated association supports the validity of the square root transformation, as it effectively minimises the confounding influence of baseline lesion size.

Figure 3 visually illustrates GA progression using concentric circles, where lesion area is depicted as expanding rings across timepoints. Panel a shows the mean lesion size of 31 eyes that completed the study, while Panel b presents individual eye trajectories. Larger lesions generally progress faster, though this is not consistent across all cases, highlighting individual variability. The linear radial expansion over time supports the square root transformation, as it reflects the geometric relationship between radius and area, validating its use in modelling GA progression.

**FIGURE 3:**
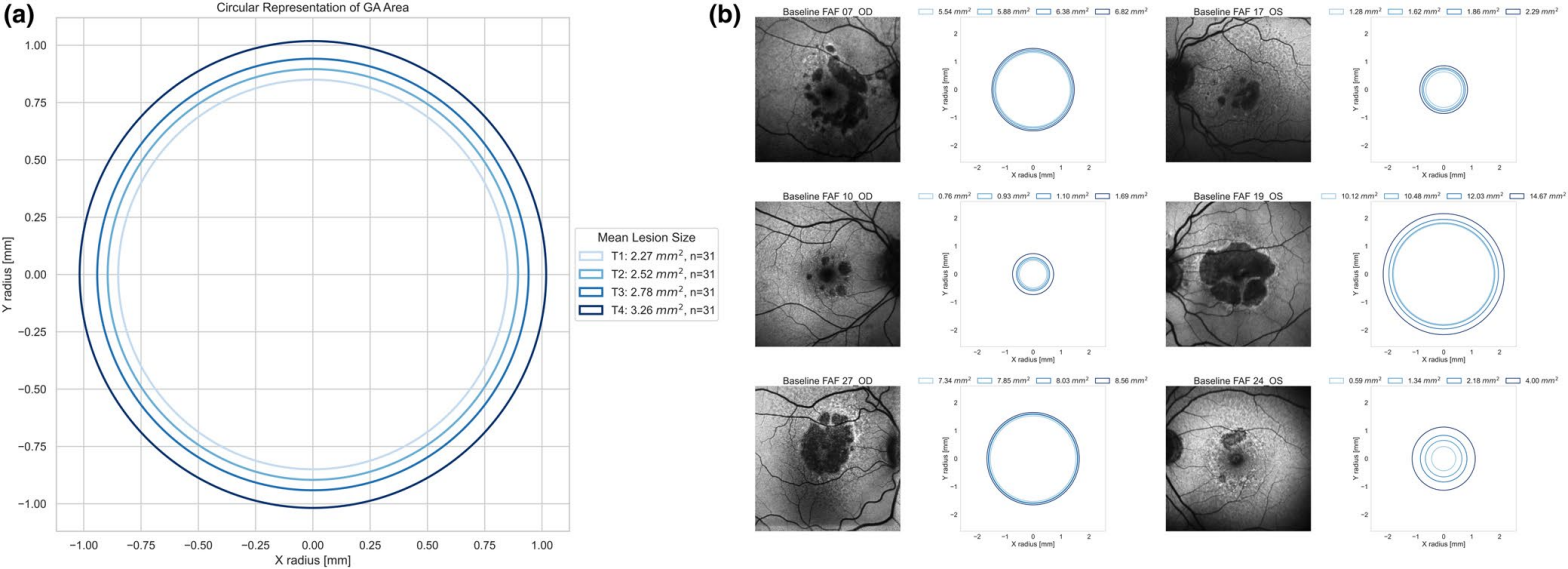
Visualisation of geographic atrophy area progression. (a) Mean lesion size across all eyes that completed the four study visits (*n* = 31), represented as concentric circles at each visit. (b) Individual eye‐level progression for individual eyes, illustrating lesion expansion over time. Circle radii increase linearly, reflecting the square root relationship between radius and area.

The first set of mixed‐effects regression models, using square‐root‐transformed GA area as the outcome, assessed the influence of various clinical covariates and their interaction with time. In the univariable mixed‐effects models, none of the tested covariates except one showed a significant influence on progression. Systolic blood pressure showed a beta of 0.003, indicating a very mild influence on progression (*p* = 0.045). Most main effects were non‐significant; smoking pack‐years was the only variable associated with a larger baseline lesion size (*p* = 0.024). In the multivariable linear mixed‐effects regression model, smoking pack‐years emerged as the only statistically significant predictor among the fixed effects. This suggests an association with a larger GA lesion size (square‐root transformed (*p* = 0.017); non‐transformed model (*p* = 0.012)) and faster GA progression (only non‐transformed model (*p* = 0.038)). The other clinical factors, including sex, BMI, lens status, blood pressure, axial length and BCVA did not show significant main or interaction effects as shown in Table 2.

**TABLE 2:** Clinical parameter‐based univariable and multivariable linear mixed‐effects regression models of square‐root‐transformed GA area progression.

Characteristic	Univariable			Multivariable		
	Beta	95% CI	*p*‐value	Beta	95% CI	*p*‐value
(Intercept)	1.333	0.99, 1.67		1.2	0.31, 2.0	0.011*
Years	0.344	0.26, 0.43	**<0.001**	0.37	0.07, 0.68	0.021*
Gender						
Male	–	–	–	–	–	–
Female	0.272	−0.34, 0.88	0.432	0.21	−0.56, 0.98	0.6
Years * Gender: female	−0.002	−0.18, 0.18	0.987	−0.02	−0.29, 0.26	>0.9
BMI	−0.039	−0.13, 0.05	0.399	−0.13	−0.28, 0.01	0.07
Years * BMI	<0.001	−0.02, 0.02	0.986	0.01	−0.04, 0.06	0.8
Smoking status						
Never	–	–	–	–	–	–
Ex‐smoker	−0.499	−1.27, 0.27	0.191	0.13	−0.92, 1.2	0.8
Smoking Status: smoker	−0.169	−1.16, 0.83	0.733	−0.14	−1.4, 1.2	0.8
Years * ex‐smoker	−0.102	−0.30, 0.09	0.298	−0.04	−0.41, 0.32	0.8
Years * smoker	−0.074	−0.33, 0.18	0.569	−0.16	−0.61, 0.30	0.5
Smoking PY	0.013	0.00, 0.03	0.024*	0.03	0.01, 0.05	0.017*
Years * Smoking PY	0.001	−0.00, 0.00	0.343	0	0.00, 0.01	0.5
Lens status						
Pseudophakic	–	–	–	–	–	–
Immature cataract	−0.013	−1.22, 1.20	0.983	0.31	−1.3, 1.9	0.7
Lens status: mild cataract	−0.448	−1.41, 0.51	0.349	−0.46	−1.7, 0.75	0.4
Years * immature cataract	−0.069	−0.38, 0.24	0.635	0.1	−0.44, 0.65	0.7
Years * mild cataract	0.088	−0.17, 0.35	0.478	0.12	−0.30, 0.54	0.5
Age at consent	0.03	−0.03, 0.09	0.322	0.02	−0.05, 0.10	0.5
Years * Age at consent	0.006	−0.01, 0.02	0.438	0.01	−0.01, 0.04	0.3
BCVA	−0.001	−0.00, 0.00	0.55	0	0.00, 0.00	0.6
Years * BCVA	0.002	−0.00, 0.01	0.521	0	0.00, 0.01	0.4
Axial length	0.02	−0.03, 0.07	0.462	0.01	−0.04, 0.07	0.6
Years * Axial length	−0.008	−0.02, 0.00	0.087	−0.01	−0.02, 0.00	0.088
SBP	−0.0001	−0.00, 0.00	0.834	0	0.00, 0.00	0.9
Years * SBP	0.003	0.00, 0.01	0.045*	0	0.00, 0.01	0.075

Abbreviations: BCVA, best corrected visual acuity; BMI, body mass index; CI, confidence interval; PY, pack years; SBP, systolic blood pressure.

The results of the univariable and multivariable mixed‐effects models, including imaging‐parameter‐based covariates and using square‐root transformed GA lesion size, are shown in Table 3. In the univariable analysis, faster GA progression was observed in eyes with intermediate AMD in the fellow eye (*p* = 0.027), and perilesional hyperautofluorescence was associated with larger baseline lesions (*p* = 0.015). In the multivariable mixed‐effect models, faster progression in eyes with intermediate AMD fellow‐eye was found again (*p* = 0.041). Perilesional hyperautofluorescence (*p* = 0.044) and additionally multifocality of the lesions (*p* = 0.038) were significantly associated with larger baseline GA lesions. Other covariates were not significant predictors of baseline size or progression.

**TABLE 3:** Imaging parameter‐based univariable and multivariable linear mixed‐effects regression model of square‐root‐transformed GA area progression.

	Univariable			Multivariable		
	Beta	95% CI	*p*‐value	Beta	95% CI	*p*‐value
(Intercept)	1.333	0.99, 1.67		1.4	0.52, 2.2	0.003*
Years	0.344	0.26, 0.43	<0.001*	0.21	0.00, 0.43	0.049*
Partner eye diagnosis						
Study Eye	–	–	–	–	–	–
iAMD	−0.254	−1.33, 0.82	0.647	−0.25	−1.6, 1.1	0.7
nAMD	0.425	−0.49, 1.34	0.373	0.12	−0.93, 1.2	0.8
GA	0.212	−0.71, 1.13	0.656	0.2	−0.86, 1.3	0.7
Years * iAMD	0.319	0.06, 0.58	0.027*	0.35	0.02, 0.69	0.041*
Years * nAMD	0.063	−0.16, 0.28	0.58	0.01	−0.26, 0.27	>0.9
Years * GA	0.165	−0.06, 0.39	0.17	0.16	−0.12, 0.43	0.2
RPD						
No	–	–	–	–	–	–
Yes	−0.235	−0.98, 0.52	0.544	−0.12	−1.1, 0.84	0.8
Years * yes	0.136	−0.05, 0.32	0.156	0	−0.24, 0.25	>0.9
Perilesional hyperautofluorescence						
No	–	–	–	–	–	–
Yes	0.674	0.17, 1.18	0.015*	0.61	0.02, 1.2	0.044*
Years * yes	0.056	−0.06, 0.17	0.352	0.12	−0.01, 0.25	0.069
Fovea involving GA						
No	–	–	–	–	–	–
Yes	0.2	−0.41, 0.80	0.522	0.11	−0.54, 0.77	0.7
Years * yes	−0.027	−0.17, 0.12	0.711	−0.1	−0.26, 0.06	0.2
Multifocality						
No	–	–	–	–	–	–
Yes	−0.349	−0.83, 0.13	0.169	−0.69	−1.3, −0.04	0.038*
Years * yes	−0.033	−0.15, 0.08	0.567	−0.04	−0.18, 0.11	0.6
Number of lesions	0.023	−0.08, 0.13	0.666	0.1	−0.03, 0.23	0.12
Years * Number of lesions	−0.000	−0.03, 0.03	0.993	0.01	−0.02, 0.04	0.6

Abbreviations: CI, confidence interval; GA, geographic atrophy (not included as a study eye); iAMD, intermediate age‐related macular degeneration; nAMD, neovascular AMD; RPD, reticular pseudodrusen.

## 4 DISCUSSION

In this study, we analysed the progression of GA area based on FAF images annotated using the RegionFinder tool. Three trained graders longitudinally segmented GA lesions from the OMEGA cohort, enabling both eye‐level and lesion‐level progression analyses. Across 37 eyes from 30 patients, we observed consistent and measurable progression of GA, with an average increase in total GA area of 1.16 mm^2^/year. The square root‐transformed progression rate was 0.34 mm/year. No lesion confluence was detected, but five eyes developed new lesions over the 48‐week period, and the average lesion count per eye slightly increased. These findings support the role of GA area as a reliable endpoint in clinical trials and contribute to the growing body of natural history data critical for evaluating emerging therapeutics. In addition, we present an intuitive visualisation for GA area progression by mapping its area over time as expanding concentric circles.

Our analysis confirmed that larger baseline lesions tended to progress more rapidly, with a strong correlation (*R* ^2^ = 0.74) observed between initial size and growth rate in the non‐transformed model. This association was markedly reduced (adjusted *R* ^2^ = 0.16) following square‐root transformation, confirming its use in reducing size‐related bias (Feuer et al., 2013; Sadda et al., 2018). When compared with prior research, our observed progression rate falls within the lower mid‐range of existing literature (Domalpally et al., 2016; Fleckenstein et al., 2018; Jaffe et al., 2015; Mata et al., 2013). A meta‐analysis by Fleckenstein et al. reported GA growth rates ranging from 0.53 to 2.6 mm^2^/year, with a median of 1.78 mm^2^/year (Fleckenstein et al., 2024).

Multivariable modelling showed that most systemic or ocular parameters, such as age, sex, BMI, lens status, axial length and BCVA, did not significantly impact GA growth. Only smoking pack‐years showed statistically significant effects across different models regarding the clinical parameters. The association with smoking is consistent with previously reported mechanisms involving antioxidant depletion, complement activation and vascular alterations (Velilla et al., 2013; Woodell & Rohrer, 2014). It was previously shown that smokers have an earlier onset of advanced AMD and develop GA 3.5 years earlier than never‐smokers, independent of major genetic risk loci such as ARMS2/HTRA1 (Khan et al., 2006; Seddon et al., 2020). Overall, these results confirm that GA progression is predominantly time‐driven, with limited influence from baseline clinical characteristics.

Our analysis of imaging‐based parameters revealed that perilesional hyperautofluorescence and lesion multifocality were associated with larger baseline GA areas, which supports earlier findings indicating a positive correlation between the area of increased FAF and multifocality of the lesions with larger GA lesions (Schmitz‐Valckenberg et al., 2006, 2016). The fovea involvement in GA and RPD did not show any statistically significant findings, yet the trend for both findings goes in the same direction as the previous literature. The presence of RPD predicts a faster rate of GA enlargement in AMD, while fovea‐involvement of the GA lesion is associated with slower progression (Agrón et al., 2023; Schmitz‐Valckenberg et al., 2016). Interestingly, in our study, the presence of intermediate AMD in the fellow eye was associated with faster GA progression, whereas previous literature has commonly identified fellow‐eye late AMD as a marker of accelerated growth (Fleckenstein et al., 2011; Grassmann et al., 2015). Our observation may indicate that inter‐eye asymmetry such as the presence of advanced GA in one eye and intermediate AMD in the fellow eye could reflect a more aggressive disease trajectory in certain individuals and that there remain difficulties in predicting individual disease trajectories based on clinical and imaging‐based covariates alone. Additional insights could be gained through the integration of multimodal imaging, functional assessments and broader systemic evaluations, including genetic profiling, laboratory testing and investigation of nutritional supplements.

There are several limitations to consider. First, the follow‐up duration was limited to 48 weeks, which may not fully capture the long‐term dynamics of lesion coalescence or plateauing in growth seen in advanced stages. The dropout of six eyes between baseline and final follow‐up could introduce attrition bias, although the remaining cohort remained similar in baseline characteristics. Future work could include perimeter‐adjustment in addition to the square‐root transformation for the GA areas (Shen et al., 2021, 2023). The RegionFinder tool, while standardised, may miss subtle low‐signal margins of atrophic regions, potentially underestimating lesion size in some eyes. Lastly, the relatively small sample size limits power for detecting effect sizes in clinical covariates. Larger studies could take into account subgroups of autofluorescence patterns and combine their analysis with genetic information (Deng et al., 2022; Holz et al., 2007; Schwartz et al., 2025). Furthermore, additional modalities such as FAF lifetime imaging, or analytical approaches such as artificial intelligence‐based photoreceptor‐RPE loss ratios, could be incorporated to enable pixel‐level prediction (Egger et al., 2025; Hammer & Meller, 2023). Further analysis of localised structural changes and analysing regional changes may help to refine prognostic models and patient‐tailored risk stratifications.

Despite these limitations, the findings of the OMEGA study can be compared to similar clinical populations with non‐exudative AMD (Heier et al., 2023; Khanani et al., 2023; Patel et al., 2023). The inclusion of eyes across a spectrum of lesion sizes, and the use of FAF, enhance external validity. Furthermore, the demonstrated consistency in growth patterns across individual eyes, combined with the low impact of clinical covariates, suggests that GA enlargement follows a relatively uniform natural trajectory within individual eyes. These insights are valuable for clinicians and researchers designing monitoring schedules or evaluating the effectiveness of emerging interventions (Broadbent et al., 2024; Spaide & Vavvas, 2023).

## 5 CONCLUSION

In conclusion, our findings confirm that lesion progression is both size‐dependent and subject to considerable inter‐individual variability. Larger baseline lesions consistently exhibited more rapid growth, underscoring a strong size‐dependency in progression dynamics. Perifoveal hyperautofluorescence and multifocal lesions were associated with large GA areas per eye, and intermediate AMD in the fellow eye was associated with a faster progression. Substantial variability in progression rates suggests that additional biological and environmental factors influence disease trajectories. Among these, smoking pack years emerged as a significant contributor. These results point towards the importance of considering lesion size, lesion configuration and smoking history when assessing risk and tailoring follow‐up strategies in clinical and research settings.

## CONFLICT OF INTEREST STATEMENT

Philippe Valmaggia: Recipient of Heidelberg Engineering and Bayer, Funding from Swiss National Science Foundation (Grant 323530_199395), Swiss Academy of Medical Sciences (YTCR 43/23) and Janggen‐Pöhn Foundation. Hanna Zuche: Recipient of Bayer and Apellis. Kristina Pfau: Consultant of Daiichi Sankyo. Andrea Giani: Employee of Boehringer Ingelheim. Marieh Esmaeelpour: Employee of Boehringer Ingelheim. Taffeta C. Yamaguchi: Employee of Boehringer Ingelheim. Christian Prünte: Consultant of Alcon, Bayer, Novartis, Oertli, Opharmic, Roche, and Tavo; recipient of Alcon, Bayer and Oertli. Hendrik P.N. Scholl: Swiss National Science Foundation (Project funding: ‘Developing novel outcomes for clinical trials in Stargardt disease using structure/function relationship and deep learning’ #310030_201165) and the Wellcome Trust (PINNACLE study). Dr. Scholl is chief medical officer of Belite Bio, but this work is performed outside of his activities for Belite Bio and not endorsed by Belite Bio. Dr. Scholl is the director of Bioptima AG. He is a member of the Scientific Advisory Board of AIER Hospital Group, Boehringer Ingelheim Pharma GmbH & Co, Janssen Research & Development, LLC (J&J Innovative Medicine), Kerna Ventures, Okuvision GmbH and Tenpoint Therapeutics. Dr. Scholl is a member of the Investment Advisory Board of Droia NV. Dr. Scholl is a member of the Data Monitoring and Safety Board/Committee of ViGeneron (NCT06291935) and an adviser of the Steering Committee of Novo Nordisk (FOCUS trial; NCT03811561). Nicolas Feltgen: Consultant of Roche, Bayer, Apellis. Leopold Schmetterer: Consultant of Boehringer Ingelheim Pharma GmbH & Co, Thea Pharma, Centervue Spa, Funding from Johnson&Johnson. Maximilian Pfau: Recipient of Novartis, Consultant of: Janssen Pharmaceutica, Apellis, Daiichi Sankyo, Funding from Apellis. Peter M. Maloca: P.M.M. is a consultant at Roche (Basel, Switzerland), Zeiss Forum and holds intellectual property for machine learning at MIMO AG and VisionAI, Switzerland.

## Data Availability

This article includes the data generated or analysed during the study and additional data can be found in other OMEGA study reports. For further information, please contact the corresponding author.
